#  DeviceEditor visual biological CAD canvas

**DOI:** 10.1186/1754-1611-6-1

**Published:** 2012-02-28

**Authors:** Joanna Chen, Douglas Densmore, Timothy S Ham, Jay D Keasling, Nathan J Hillson

**Affiliations:** 1 Fuels Synthesis Division, Joint BioEnergy Institute, Emeryville, CA 94608, USA; 2 Physical Bioscience Division, Lawrence Berkeley National Lab, Berkeley, CA 94720, USA; 3 Department of Electrical and Computer Engineering, Boston University, Boston, MA 02215, USA; 4 Sandia National Laboratories, Livermore, CA 94550, USA; 5 Department of Chemical & Biomolecular Engineering, University of California, Berkeley, CA 94720, USA; 6 Department of Bioengineering, University of California, Berkeley, USA

**Keywords:** bioCAD, Visual design abstraction, Correct-by-construction design, Design specification rules, Combinatorial library, DNA assembly

## Abstract

**Background:**

Biological Computer Aided Design (bioCAD) assists the *de novo *design and selection of existing genetic components to achieve a desired biological activity, as part of an integrated design-build-test cycle. To meet the emerging needs of Synthetic Biology, bioCAD tools must address the increasing prevalence of combinatorial library design, design rule specification, and scar-less multi-part DNA assembly.

**Results:**

We report the development and deployment of web-based bioCAD software, DeviceEditor, which provides a graphical design environment that mimics the intuitive visual whiteboard design process practiced in biological laboratories. The key innovations of DeviceEditor include visual combinatorial library design, direct integration with scar-less multi-part DNA assembly design automation, and a graphical user interface for the creation and modification of design specification rules. We demonstrate how biological designs are rendered on the DeviceEditor canvas, and we present effective visualizations of genetic component ordering and combinatorial variations within complex designs.

**Conclusions:**

DeviceEditor liberates researchers from DNA base-pair manipulation, and enables users to create successful prototypes using standardized, functional, and visual abstractions. Open and documented software interfaces support further integration of DeviceEditor with other bioCAD tools and software platforms. DeviceEditor saves researcher time and institutional resources through correct-by-construction design, the automation of tedious tasks, design reuse, and the minimization of DNA assembly costs.

## Background

The development of bioCAD software is paramount to our future capacity to rapidly design increasingly complex biological systems for the predictable and reproducible production of biofuels and bio-based chemicals [1]. When considering a DNA construction task, researchers must choose from a rapidly expanding list of candidate gene orthologs and expression systems. BioCAD tools (reviewed in [2-4]) make it possible to automatically query parts repositories for putative design components [5] and model the performance of candidate component combinations [6-9]. These software tools can also address design workflow bottlenecks by providing canvases for abstractly visualizing and arranging genetic components [10] and automating the design and execution of the DNA assembly process [11,12] (reviewed in [13,14]).

However, despite the growing utility of bioCAD software, three critical design automation needs within the Synthetic Biology community remain unmet: 1) software integration, 2) combinatorial library design visualization, and 3) user-specifiable design rules. First and foremost, the end-to-end design process is crippled by the lack of integration among individual software tools that specialize in modelling, DNA assembly, or genetic component (e.g., ribosomal-binding site (RBS) [15]) design. It is hoped that emerging data exchange standards such as the Synthetic Biology Open Language (SBOL) [16], along with open and well-documented software interfaces, will enable future bioCAD platforms and minimize tool redundancy. Second, combinatorial libraries of fusion proteins and metabolic pathways have become increasingly utilized for optimizing biofuel and bio-based chemical production [17-19], yet no visual bioCAD tools currently support the combinatorial library design process. Algorithms have been developed to automate the enumeration of all combinations of genetic components that meet a given set of design specifications [12,20], but the input of the DNA sequence information and the execution of these algorithms is not visually intuitive. More useful would be an interface that captures a familiar design workflow, such as the ubiquitous dry-erase whiteboard, to facilitate the spatial arrangement of components to be combined. Third, while bioCAD tools have been developed to visualize specification-compliant designs [21] or exploit composition grammars to guide the visual arrangement of parts [10], the underlying specifications and grammars must be defined within a programming-like language [20,22] or remain opaque by being neither viewable nor modifiable by the user [10].

Towards addressing these unmet design needs, we have developed DeviceEditor, a bioCAD canvas that enables researchers to spatially organize abstractions of biological components. DeviceEditor assists the aggregation and arrangement of the DNA sequences of genetic components (e.g., ribosomal-binding sites, promoters and terminators, and metabolic pathway genes) to be assembled towards a desired functionality. DeviceEditor ensures that designs are "correct-by-construction", because within its confines researchers are prevented from performing invalid operations (e.g. referencing DNA base-pair 500 within a 100 base-pair sequence). To the best of our knowledge, DeviceEditor is the first bioCAD tool that visualizes combinatorial DNA library design, provides a graphical user interface for the creation and modification of design specification rules, and is directly integrated with scar-less multi-part DNA assembly design automation. Taken together, these innovations benefit researchers and their institutions through correct-by-construction design, the automation of tedious tasks, design reuse, and the minimization of DNA assembly costs.

## Results

**Figure 1:**
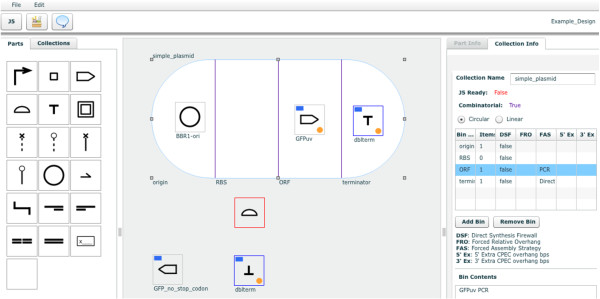
**DeviceEditor design canvas**. Screenshot of the browser-based DeviceEditor user interface [24]: (top left) buttons for activating the j5 controls dialog box and setting DeviceEditor properties, (left panel) palette of standardized SBOLv part icons, (center) drag-and-drop design canvas with part icons and a collection object (white oval with vertical lines demarking bins), and (right panel) information detail for the selected part or collection.

The DeviceEditor bioCAD canvas provides a web-based visual design environment (Figure 1) that mimics the familiar whiteboard design process practiced in biological laboratories. An online user's manual [23] provides an introduction to bioCAD, an overview of DeviceEditor functionality, and step-by-step how-to video demonstrations.

### DeviceEditor design process

**Figure 2:**
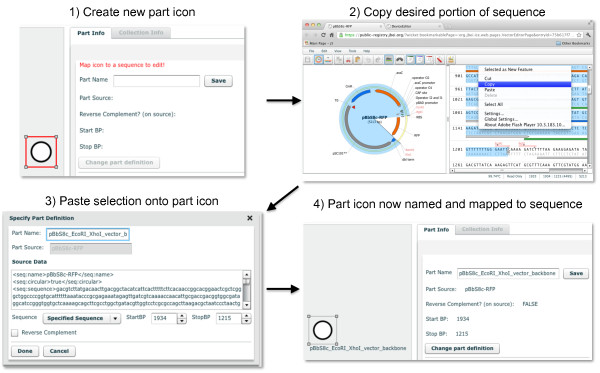
**Mapping a DeviceEditor part icon to an annotated DNA sequence**. A new part icon on the design canvas (top left) is created by clicking on the desired SBOLv icon (here "Origin of Replication") in the left panel of the user interface (Figure 1). At this point, a DNA sequence has yet to be mapped to the part icon. In a separate browser-tab or software application, the desired portion of a DNA sequence (here the pBbS8c-*rfp *backbone [27] spanning from *Xho*I to *EcoR*I) is selected and copied (top right) to the clipboard. Third-party software (here VectorEditor) may embed meta-data (including jbei-seq format [28] sequence data) into the clipboard along with the plain-text DNA sequence selection (see Methods). Returning to DeviceEditor, the copied DNA sequence is pasted (mapped) from the clipboard onto the part icon. Clipboard meta-data provides DeviceEditor with the selected start and stop base pairs (here 1934 to 1215) within the circular source sequence, along with the source's name (here pBbS8c-*rfp*), entire sequence, and feature annotations (displayed in the "Source Data" field; bottom left). If the third-party software (e.g. ApE [29]) does not embed this meta-data, the sequence annotations are not transferred to DeviceEditor, and the user must specify the source name and the selected start and stop base-pairs within the copied sequence. The user may alternatively map a Genbank-format sequence file to the part icon, which preserves the source name and feature annotations. The name for the part icon (here "pBbS8c_EcoRI_XhoI_vector_backbone") is specified, along with whether the part is associated with the reverse complement of the selected sequence. The "Done" button is clicked, the part icon has now been named, and the desired annotated DNA sequence has been mapped to the part icon (bottom right).

**Figure 3:**
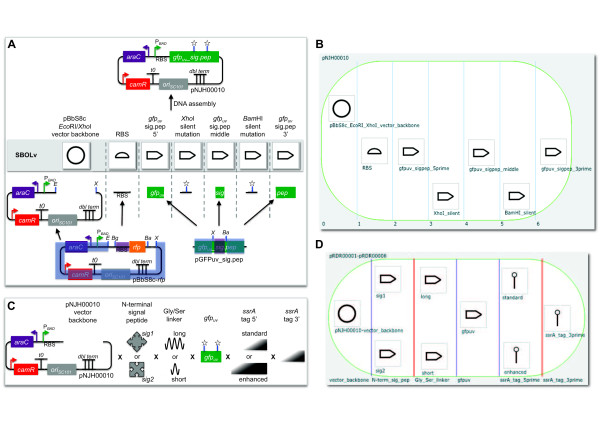
**Example biological designs rendered on the DeviceEditor canvas**. (A) pNJH00010 [12] consists of seven components: the pBbS8c-*rfp *backbone spanning from *Xho*I to *EcoR*I (blue highlight, bottom left), the RBS sequence from pBbS8c-*rfp *(purple highlight, bottom left), *gfp_uv _*from its 5' end to its *Xho*I site (blue highlight, bottom right), a silent mutation in *gfp_uv_*'s *Xho*I site (star), *gfp_uv _*between its *Xho*I and *BamH*I sites (purple highlight, bottom right), a silent mutation in *gfp_uv_*'s *BamH*I site (star), and *gfp_uv__sig.pep *from its *BamH*I site to its 3' end (blue highlight, bottom right). These components are arranged from left to right in their 5' to 3' order in pNJH00010 (top). The corresponding SBOLv icon is presented immediately above each component. (B) To reconstitute the design in (A), each of the component sequences is mapped to a part icon (as in Figure 2), and arranged from left to right in 5' to 3' order as in (A) in a 7-bin collection object (white oval with vertical blue lines demarking bins), with each part icon in its own bin. This DeviceEditor design has been saved in Additional file 1. (C) The combinatorial design for plasmids pRDR000001-pRDR000008 [12] consists of nine components, including the pNJH00010 backbone, two N-terminal signal peptides (*sig1 *and *sig2*), two Gly/Ser linkers (long and short), the *gfp_uv _*mutant from pNJH00010, two 5' *ssrA *tags (standard and enhanced), and a 3' *ssrA *tag. These components are arranged from left to right from 5' to 3', with interchangeable components arranged from top to bottom. (D) Each of the component sequences in (C) is mapped to a part icon, and these part icons are then arranged from left to right as in (C) in a 6-bin collection object, with interchangeable part icons in the same bin. Each bin, now demarcated with purple lines indicating a combinatorial design, is then named according to the category of parts it contains (bottom) This DeviceEditor design has been saved in Additional file 2.

To begin the process, the genetic components or biological "parts" that will comprise the design are defined. This is accomplished by first selecting a standardized icon from the Synthetic Biology Open Language Visualization extension (hereafter SBOLv) [25] palette to represent a given component (e.g., promoter, 5' UTR, terminator). If no icon in the SBOLv palette fully captures the essence of a part (e.g., the pBbS8c-*rfp *backbone consists of more than just an "Origin of Replication", Figure 2), the most appropriate option or a blank generic icon can be selected. While visually evocative, from the outset the icons do not contain actual information. A DNA sequence is then mapped to the part icon (Figure 2), either copied from third party software or retrieved from a sequence file. DNA sequences in DeviceEditor do not need to be "packaged" in any particular format, such as the BioBricks format [26]. Each part is then set to either "Forward" (default) or "Reverse" to indicate the desired orientation of the part in the resulting construct. Once all of the desired component part icons have been defined, they are arranged from left to right in a "collection" to match their 5' to 3' order in the target DNA construct (Figure 3A, B; Additional file 1). For combinatorial designs, interchangeable component icons are arranged in the same vertical collection "bin" (Figure 3C, D; Additional file 2). The design is then specified to produce a "Circular" (default) or "Linear" DNA construct (Figure 1, top right).

**Figure 4:**
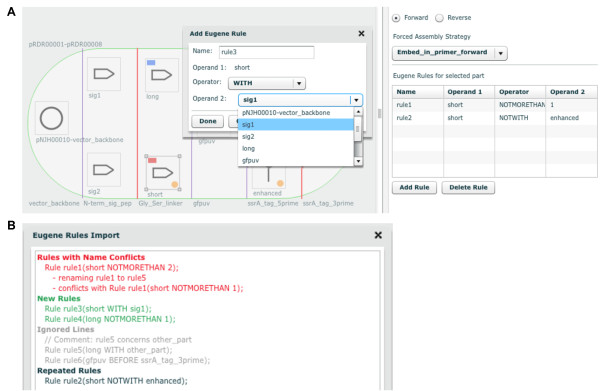
**Adding Eugene design specification rules**. (A) Graphical user interface for creating and modifying rules. A part icon (here "short", bottom left) on the design canvas is clicked, followed by the "Add Rule" button in the right panel of the user interface (bottom right). The name for the rule (here "rule3") is specified, and one of three Eugene operators (NOTMORETHAN, WITH or NOTWITH) is selected (here "WITH"). For the NOTMORETHAN operator, the maximum number of times the part may be present in a single construct is specified. For the WITH or NOTWITH operators, the other part icon on the design canvas (Operand 2, here "sig1") that should or should not be present in a single construct, respectively, with the selected part icon (Operand 1, here "short") is chosen. The list of Eugene rules associated with the selected part icon is shown in the right panel of the user interface (right). Part icons with associated Eugene rules are visually identified on the design canvas by an orange circle indicator light at bottom right, and part icons with specified forced assembly strategies are distinguished with a blue (bin consensus-matching assembly strategy) or a red (bin consensus-breaking assembly strategy) rectangle indicator at top left. (B) Importing Eugene rules from a file. From the "File" pull-down menu of the user interface (Figure 1, top left), "Import Eugene Rules" is clicked and a Eugene rules file (e.g. Additional file 3) is selected. The Eugene Rules Import dialog displays imported rules in green, rules identical to current rules in black, imported rules with names conflicting with current rules displayed in red (alternative names are auto-generated for the imported rules to resolve conflicts), and ignored rules (e.g. comment lines or rules with invalid operators or operands not present in the current design) in light grey. Importing a set of Eugene rules facilitates the batch creation of multiple rules for complex designs (Figure 5).

**Figure 5:**
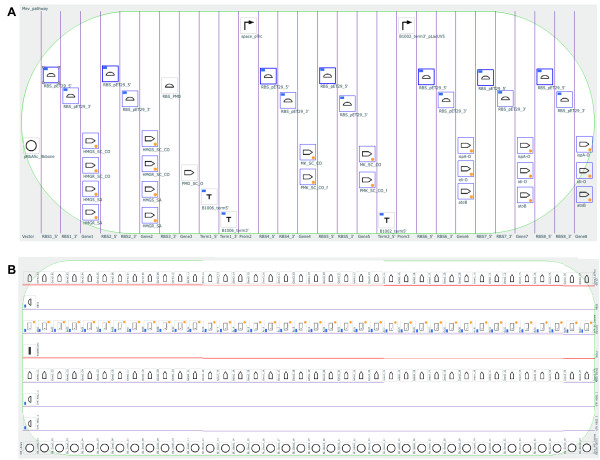
**Hypothetical combinatorial DeviceEditor designs**. (A) Metabolic pathway library: 29 bins and 19 unique part icons (several repeated through the design). Clicking on a repeated part icon, here "RBS_pET29_5'" (top left), highlights all replicates with thick blue outlines. (B) Gene over-expression library (rotated 90° counter-clockwise): 8 bins and 156 part icons (none repeated). Note that there are only 38 barcodes (sixth bin) that correspond one-to-one with the 38 vector backbones (first bin). This design allows for a single short sequencing read spanning the end of gene 2 (fourth bin) through the barcode and the beginning of gene 3 (eighth bin) to uniquely identify a plasmid combination. As a result, there is not a unique barcode required for each of the 38^3 ^= 54,872 possible plasmid combinations. Zoom in with PDF display software to improve legibility.

To limit the total number of times a part may appear in a given construct, to prevent any two parts from appearing together in the same construct, or to ensure that two given parts always appear together in the same construct, Eugene design specification rules [20] may be added. These rules can ensure that two parts always appear together in the same construct, prevent two parts from appearing together in the same construct, and limit the total number of times a part may appear in a construct. For example, if prior research demonstrated that the short linker sequence must be used with the tag *sig1 *(Figure 3C) to achieve proper GFPuv localization, Eugene rules can be specified (Figure 4; Additional file 3) to ensure that the short linker is always constructed together with *sig1*. This would eliminate two (of the eight possible) combinations that have the short linker following the tag *sig2*. For metabolic pathway library designs that vary enzyme ortholog selection and gene ordering [30,31], Eugene rules can ensure that orthologs and individual enzymes are not repeated in the same construct. The application of just 9 Eugene rules to the design shown in Figure 5A eliminates 1632 undesirable combinations that do not constitute complete pathways, out of 1728 total possible combinations. Also, the set of "desirable" combinations may evolve with additional experimental information, for example, if evidence arises that two particular enzyme orthologs do not operate well together in *E. coli*. A key point is that this additional information only requires the modification of a few design rules to update the desirable set of combinations, rather than complete re-designs. This is important because toggling a few design rules enables researchers to re-use designs as they switch between microbial hosts and as they gain experimental insight. For selection-based experiments exploiting pooled-library designs [32], Eugene rules can ensure that each vector backbone is always constructed together with its one-to-one corresponding user-specified DNA barcode (Figure 5B), facilitating and reducing the cost of sequence-identifying top performers.

**Figure 6:**
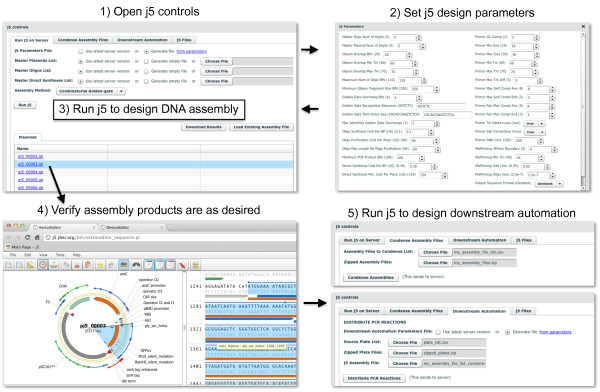
**Running j5 from within DeviceEditor**. The j5 button at the top left of the user interface (Figure 1) is clicked to open the j5 controls dialog box (top left). The j5 design parameters [12] may be customized or returned to their default values by clicking the "from parameters" link at the top right of the "Run j5 on Server" tab (top left), which opens the j5 Parameters dialog box (top right). Otherwise, the user's latest set of design parameters (stored on the j5 server) will be employed. Similarly, the user's latest lists of plasmids, oligos, and direct syntheses will be used (shown here), unless empty or alternate lists are specified. For single construct designs (Figure 3B), the assembly method may be either "SLIC/Gibson/CPEC" or "Golden-gate". For combinatorial designs (Figure 3D), the assembly method may be either "Combinatorial SLIC/Gibson/CPEC" or "Combinatorial Golden-gate" (shown here). After the "Run j5" button is clicked, DeviceEditor submits the design contained within the collection to the j5 server (see Methods) and provides links to the resulting assembled sequences. Clicking on one of these links (here "pj5_00003.gb") will open the corresponding sequence in VectorEditor (bottom left) so that it may be easily verified (here, the N-terminal signal peptide, Gly/Ser linker, *gfp_uv_*, and ssrA tag (Figure 3C, D) are confirmed to be in-frame). The "Condense Assembly Files" and "Downstream Automation" tabs in the j5 controls dialog box (bottom right) provide access to j5 downstream automation design.

To integrate physical implementation (i.e. DNA assembly) strategy into the DeviceEditor design process, the means by which the parts should be assembled together (i.e. "forced assembly strategies") may optionally be prescribed for each part (Figure 4A, top right). Part icons with forced assembly strategies are visually distinguished with a blue rectangle indicator light at the top left (Figure 4A, left). Additional aspects of DNA assembly strategy may be further customized in the "Collection Info" panel (Figure 1, right). For any given bin in a collection, a "direct synthesis firewall" may be set to "true" to prevent the extension of DNA synthesis through the assembly junction [12] to the right of the bin, as visually indicated by a red vertical line between bins (Figure 3D). The optional "forced relative overhang/overlap" position for each bin prescribes the overhang/overlap position of the assembly junction [12] to the right of the bin. Finally, the forced assembly strategy for each bin is displayed but is not directly modifiable, as it is determined by the forced assembly strategies of the parts that the bin contains. To automate DNA assembly, DeviceEditor submits the design contained within the collection to j5, a web-based software tool for designing cost-optimized, scar-less, multi-part, DNA assembly protocols [12] (Figure 6, top). Part icons peripheral to the collection are not submitted as part of the design to j5. Once users have visually verified that the desired constructs are correctly designed, DeviceEditor can also direct j5 to design downstream automation processes, such as condensing multiple assembly files and distributing PCR reactions by annealing temperature (Figure 6, bottom).

### Design process acceleration and correct-by-construction design

DeviceEditor accelerates the design process by relieving the user of tedious routine tasks. For example, sequence annotations and other meta-data are retained when sequences are copied via a compatible clipboard format (see Methods) and pasted onto part icons (Figure 2), or mapped from Genbank format sequence files, obviating the need to re-annotate sequences post-assembly. Part icons can be copied and pasted between concurrent DeviceEditor sessions, enabling the re-use of previously defined parts. Also, DeviceEditor provides hyperlink shortcuts to view j5-assembled design sequences in VectorEditor [33,34] (Figure 6) for rapid visual design feedback.

The DeviceEditor correct-by-construction design process prevents common mistakes. Within the confines of DeviceEditor, the user is not able to perform invalid design modifications. For example, icons for repeated parts are internally linked together (Figure 5A), so that when one instance of a part is modified, all instances are updated in unison, precluding the persistence of obsolete information or the introduction of inconsistencies between repeated parts. DeviceEditor's correct-by-construction features also benefit j5 DNA assembly design automation [12]. The j5 web-form interface [35] requires the user to upload several comma-separated value (CSV) input files. When manually preparing these CSV input files with spreadsheet software (e.g. Excel, Open Office), there are no safeguards against mistyping start and stop base-pair numbers, or placing a direct synthesis firewall at an unintended assembly junction. Since spreadsheet software does not constrain the user's input, j5 design parameters may be specified out of their acceptable ranges, part names may incorporate typographical errors or prohibited characters, and sequence file names may be mistakenly entered instead of sequence display IDs (a subtle, yet common point of frustration). In contrast with the manual preparation of CSV input files, the DeviceEditor interface for j5 ensures that design parameters fall within their acceptable ranges (Figure 6), validates the uniqueness and correctness of part names, automatically extracts sequence display IDs, prevents start and stop base-pair numbering mistakes (Figure 2), and visualizes the selected placement of direct synthesis firewalls (red vertical lines in Figures 3D and 5B). DeviceEditor's correct-by-construction features can optionally prevent the user from moving a part icon with a "DIGEST" forced assembly strategy to the first collection bin, as this would be problematic for downstream j5 DNA assembly design [12]. Finally, DeviceEditor pre-empts substantially increased DNA assembly costs by visually alerting the user if two parts in the same bin have disparate forced assembly strategies, which greatly limits the combinatorial re-use of assembly fragments [12]. Part icons with forced assembly strategies differing from their bin are visually distinguished by a red rectangle indicator light at top left (Figure 4A, left).

### Graphical user interface for creating and modifying Eugene biological design specification rules

Several bioCAD tools (e.g. Clotho [8,36] and GenoCAD [10,37]) harness biological specification rules and expression grammars to constrain designs, but the underlying rules and grammars are not viewable or modifiable through the design tools themselves. This can be problematic for design specification rule languages such as Eugene [20] that currently rely on name-matching for part identification, since simple typographical errors can result in referencing incorrect or non-existing parts, and identical names (e.g., "vector_backbone") for distinct parts can result in the misapplication of rules. DeviceEditor's graphical user interface for creating and modifying design specification rules (Figure 4A) and its Eugene rules file import feature (Figure 4B) prevent typographical mistakes by constraining new rules to supported operators (e.g. WITH) and operands (i.e. part icons) presently on the design canvas. Part icons associated with Eugene rules are visually identified by an orange circle indicator light at bottom right (Figure 5A). DeviceEditor also prevents the misapplication of rules by 1) not allowing distinct parts to have the same name and 2) displaying all rules that specifically apply to the selected part icon (Figure 4A), precluding the need to search through thousands of unrelated rules. While DeviceEditor currently only supports a subset of Eugene rules (NOTMORETHAN, WITH, and NOTWITH), future development will expand this list towards more complete coverage.

### Mechanisms for integration with other bioCAD software

**Figure 7:**
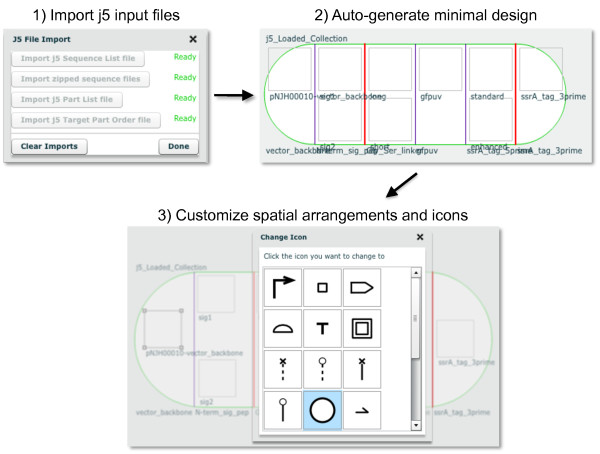
**Auto-generation of a DeviceEditor design from CSV and zipped sequence files**. Third-party software can transfer component design and (combinatorial) arrangement information to DeviceEditor via spreadsheet (CSV) and zipped sequence files (i.e. a set of j5 files, see Methods). From the "File" pull-down menu of the user interface (Figure 1, top left), "Load Design → j5 Files" will open the j5 File Import dialog box (top left). After the requisite files are selected, a minimal design is auto-generated on the DeviceEditor design canvas (top right). As evident in the screenshot taken immediately after loading the design, there is no SBOLv icon information and some of the labels may be overlapping. The precise spatial arrangements of the part icons, SBOLv icon selection, and the collection object dimensions may then be customized as desired (bottom).

While bioCAD enables the *de novo *design or selection of existing component parts to achieve a given biological activity, many of these tools (e.g. the RBS Calculator [15] and GLAMM [5]) specialize in a subset of genetic components (e.g. RBS sequences or metabolic enzyme genes) and are not directly integrated with downstream DNA assembly design automation. DeviceEditor assists the aggregation and arrangement of DNA sequences arising from disparate sources. There are three mechanisms to transfer component information from other bioCAD tools to DeviceEditor: copying and pasting (mapping) components into DeviceEditor (Figure 2), CSV and sequence files (i.e. Genbank format) (Figure 7), and DeviceEditor design files (see Methods). The latter two mechanisms are preferable for transferring multiple components to reconstitute complex designs (Figure 5), since copying and pasting is currently limited to one component at a time. CSV and sequence files are advantageous in that they are straightforward for third-party software to generate, although they convey less information (e.g. CSV files omit SBOLv icon selection information, Figure 7) than DeviceEditor design files. SBOL [16], a promising emerging data exchange standard, is an anticipated fourth mechanism for integrating DeviceEditor with other bioCAD software. Further development of DeviceEditor will support importing SBOL XML once the XML serialization of SBOL has been firmly established [38].

## Discussion

DeviceEditor standardizes visual abstraction with SBOLv icons. Consider the biological components as presented in Figure 3C, such as the centrifugal arrows for N-terminal signal peptides, and sinusoidal squiggles for Gly/Ser linkers. Those unfamiliar with these *ad hoc *visual abstractions would need to rely on the corresponding textual descriptions to determine what they actually refer to. In contrast, the DeviceEditor design canvas, as represented in Figure 3D, allows anyone familiar with SBOLv to confidently interpret the design at the granularity of the standardized icons, even without supplementary text. The use of SBOLv icons is especially compelling for rapid at-a-glance assessment of component ordering and combinatorial variations within more complex designs (Figure 5). Visual inspection of large designs may also reveal design concepts, constraints, or requirements that were previously unknown. While SBOLv icons themselves are standardized, the user may associate parts and icons in a non-standard, misleading manner. To mitigate this risk, DeviceEditor could be further developed to standardize the SBOLv icon selection process, with parts defying SBOLv categorization represented by blank generic icons. Further DeviceEditor development could also support user-added icons *en route *to their formal SBOLv incorporation.

**Figure 8:**
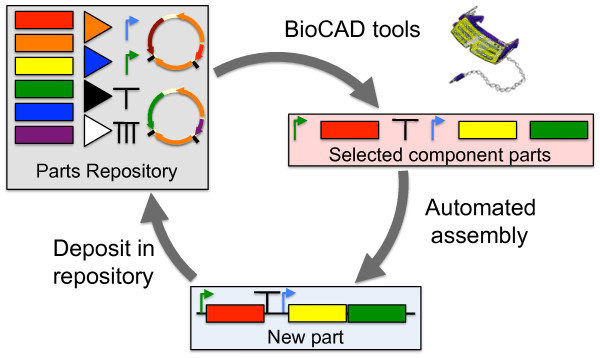
**Integrated Synthetic Biology design-implement-assay cycle**. The DeviceEditor BioCAD canvas (top right) assists the specification, selection and arrangement of biological component parts, and interfaces with upstream parts repositories (e.g. JBEI-ICE) and downstream DNA assembly automation (e.g. j5).

Data exchange standardization is vital to DeviceEditor as part of an integrated Synthetic Biology design-build-test cycle (Figure 8). Consider the simple operation of copying an annotated DNA sequence selection and pasting it onto a part icon on the DeviceEditor design canvas (Figure 2). If only plain-text DNA sequence is transferred (the most basic copy/paste operation, employed when the source application does not support copying sequence annotations), the start and stop base-pairs of the selection, the name of the source sequence, and the selection's feature annotations are all lost in the transaction. Manually supplying this missing data to DeviceEditor leaves the process susceptible to typographical errors and places a laborious sequence re-annotation burden on the user. On the other hand, transmitting a full complement of sequence meta-data precludes user-error and saves time, but demands that the software tools agree upon the structure and content of the information exchanged. While *ad hoc *data exchange adapters can be developed for open and well-documented software interfaces, as reported here for DeviceEditor, standardizing data exchange across an entire community of tools is a far more efficient approach. SBOL is one such data exchange standard, although how universally sufficient the specification will be for bioCAD has yet to be seen. For j5 in particular (and by extension DeviceEditor), sequence context (e.g. the DNA template in which a part resides) is important for safeguarding against off-target DNA oligo priming. However, SBOL currently omits this and other potentially valuable design information (such as how DNA components are to be arranged within a combinatorial collection), just as SBOLv does not adequately capture the essence of every part. The extent to which these partial omissions can and should be resolved will be an ongoing question for the Synthetic Biology community. DeviceEditor provides an environment to further investigate these issues through the targeted exploration of specific design scenarios.

DeviceEditor's correct-by-construction and design visualization features serve as the first steps towards process automation and can offer substantial time- and resource-saving benefits. Consider a Eugene rule for a part named "ispA" that is intended to limit *ispA *to one copy per construct. In Figure 5A, since the *ispA *part is instead named "ispA-O", applying this "ispA" rule would be ineffective and insufficient to prevent the construction of 112 undesired plasmid combinations that contain two or three copies of *ispA *rather than complete metabolic pathways. In DeviceEditor, these part name oversights are automatically prevented, since it would not be possible to create Eugene rules for a part named "ispA" for the design in Figure 5A. Furthermore, the lack of Eugene rule indicator lights for the repeated "ispA-O" parts on the design canvas would provide visual cues that something was amiss. Next, consider a misplacement of a direct synthesis firewall after gene 3 (eighth bin) in Figure 5B, rather than before gene 3 as desired. This mistake could result in the purchase of 1444 synthesized DNA fragments spanning the barcode to gene 3 (all pair-wise combinations of barcode and gene 3 variants) rather than the intended 76 fragments (38 barcodes plus 38 gene 3 variants) [12]. While this would be an easy mistake to make when preparing j5 input files with spreadsheet software, DeviceEditor's prominent red firewall visualization makes an incorrect firewall placement difficult to miss. Some benefits of DeviceEditor correct-by-construction design are more difficult to precisely estimate, but are nonetheless compelling given the large potential downstream risks posed by the errors they prevent. While incorrect start or stop base-pair numbering (Figure 2) and inconsistent definitions for repeated parts (e.g. the three repeats of "ispA-O" in Figure 5A) may be extremely subtle (e.g. one base-pair off), they may have dramatic negative consequences (e.g. mRNA secondary structure disruption) and require extensive detective work to resolve and incur significant time and resource losses. Copying and pasting visually selected DNA fragments from VectorEditor in to DeviceEditor (Figure 2), along with internally linked repeated parts (Figure 5A), pre-empt these costly mistakes. These case-studies provide but a few representative design scenarios where DeviceEditor can offer significant frustration, time and cost savings.

## Conclusions

BioCAD tools assist the *de novo *design or selection of existing biological component parts to achieve a specified function, as part of an integrated design-build-test Synthetic Biology cycle (Figure 8). The DeviceEditor bioCAD canvas provides a web-based SBOLv-standardized visual design environment (Figure 1) that mimics the intuitive whiteboard design process practiced in biological laboratories. DeviceEditor liberates users from DNA base-pair level design, enabling a functional level of visual abstraction that facilitates rapid prototyping. DeviceEditor adds significant value to the design process through automating routine yet tedious tasks, asserting correct-by-construction design, and providing integration with downstream DNA assembly design automation tools like j5. On-going DeviceEditor development aims to facilitate submission of assembled designs to databases, such as the JBEI-ICE [39] parts repository, yet another time-saving benefit for the user. DeviceEditor's open and documented interfaces support further development efforts towards integration with expression grammar-checking tools (e.g. GenoCAD [10]) and specialized design tools (e.g. the RBS Calculator [15] and GLAMM [5]).

## Methods

### DeviceEditor software license and availability

DeviceEditor is available at no cost to non-commercial (e.g. academic, non-profit, or government) users, under a Lawrence Berkeley National Lab end-user license agreement [40]. The software is available through the public j5 web-server [41], and is also available for download upon request. Commercial use is available through the Technology Transfer Department of Lawrence Berkeley National Laboratory (ttd@lbl.gov).

### DNA sequence availability

DNA sequences (pGFPuv_sig.pep, pBbS8c-*rfp*, pNJH00010 and pRDR00001-pRDR00008), along with their associated information (annotated Genbank-format sequence files, DeviceEditor design files, and j5 DNA assembly design files, where appropriate) have been deposited in the public instance of the JBEI Registry [39].

### DeviceEditor software implementation

DeviceEditor is web-based, available across computer platforms via a common web-browser interface (Figure 1), and as such does not require the user to install or update the software. Mediawiki software [42] coupled with a PostgreSQL database [43] serves to automate the creation and maintenance of user accounts on the public j5 web-server [41]. A sequence meta-data clipboard format developed at JBEI enables users to copy annotated DNA sequences from software supporting the format and paste them onto DeviceEditor part icons (Figure 2). DeviceEditor interacts with j5 (Figure 6, right) through j5's XML-RPC web-services interface [12]. A server-side Perl-CGI [44] script provides an interface for displaying DeviceEditor-designed assembled sequence files with VectorEditor stand-alone software [33] (Figure 6, bottom left). DeviceEditor utilizes the Adobe Flex [45], Degrafa declarative graphics [46], and PureMVC [47] programming frameworks, and draws upon the AS3 Zip [48], flex-object-handles [49], as3corelib [50], and as3-rpclib [51] software libraries. Circus Ponies Notebook software [52] was used to compose and generate the online user's manual, and QuickTime software [53] was used to create the software video demonstrations.

To enable third-party software developers to integrate their software with DeviceEditor, the specifications for the sequence meta-data clipboard format and the XML schema for DeviceEditor design files are documented in the user's manual [54]. Similarly, the specifications for j5 CSV and zipped sequences input files (Figure 7) are documented in the j5 user's manual [55].

## Abbreviations

BioCAD: Biological computer-aided design; RBS: Ribosomal-binding site; SBOL: Synthetic biology open language; SBOLv: SBOL visualization extension; CSV file: Comma-separated value file; XML file: Extensible markup language file.

## Competing interests

The authors declare competing financial interests in the form of pending software licenses whose values may be affected by the publication of this article.

## Authors' contributions

JC, DD, TSH and NJH designed the software. JC, DD and TSH developed the software. JC and NJH wrote the software user's manual. NJH created the software demonstration video tutorials. JC, DD, JDK and NJH wrote the manuscript. All authors read and approved the final manuscript.

## Supplementary Material

Additional file 1 **pNJH00010.xml - DeviceEditor design file (.xml) for pNJH00010The DeviceEditor design file for the example shown in Figure **3B. Click here for file

Additional file 2 **pRDR00001-8.xml - DeviceEditor design file (.xml) for pRDR00001-8The DeviceEditor design file for the example shown in Figure **3D. Click here for file

Additional file 3 **pRDR00001-8.eug - Eugene rules file (.eug) for pRDR00001-8The Eugene rules file for the example shown in Figure **4B. Click here for file
